# Growth Promotion of Yunnan Pine Early Seedlings in Response to Foliar Application of IAA and IBA

**DOI:** 10.3390/ijms13056507

**Published:** 2012-05-24

**Authors:** Yulan Xu, Yuemin Zhang, Yunfei Li, Genqian Li, Daiyi Liu, Minchong Zhao, Nianhui Cai

**Affiliations:** 1 College of Biological Science, Technology of Beijing Forestry University, Beijing 100083, China; E-Mails: xvyulan@163.com (Y.X.); liyunfei100@163.com (Y.L.); 2 Key laboratory for Forest Resources Conservation and Use in the Southwest Mountains of China, College of Forestry, Southwest Forestry University, Kunming 650224, China; E-Mails: Zhyyuemin@163.com (Y.Z.); liudaiyi2008@163.com (D.L.); minchong27@hotmail.com (M.Z.); cainianhui@sohu.com (N.C.)

**Keywords:** exogenous IAA and IBA, foliar application, seedling growth promotion, Yunnan pine (*Pinus yunnanensis* Franch.)

## Abstract

A field experiment was conducted using a 3 × 3 orthogonal regression design to explore the growth promotion of one-year-old Yunnan pine seedlings (*Pinus yunnanensis* Franch.) in response to foliar application of IAA (indole-3-acetic acid) at rates of 0, 200 and 400 mg·L^−1^ and IBA (indole-3-butyric acid) at rates of 0, 200 and 400 mg·L^−1^ in order to promote the growth during the seedlings’ early stage. The experiment was conducted at the Lufeng Village Forest Farm of Yiliang County in Kunming, Yunnan, China. The results showed that IAA and IBA were effective in growth promotion of Yunnan pine seedlings. The response of both growth increment and biomass accumulation to the concentration of IAA and IBA can be modeled using a bivariate surface response, and each growth index had a peak value. Growth indexes increased with the increase of the dosage of photohormones before reaching a peak value, and then decreased. The different growth indexes had various responses to the concentrations and ratio of IAA and IBA. The foliar application of IAA in combination with IBA showed the largest improvement on the biomass of the needles, followed by stems and roots. The higher ratio of IAA promoted stem diameter growth, root system development and biomass accumulation in the needles, while a higher ratio of IBA contributed to height growth and biomass accumulation in the stem. Based on the auxin effect equations on the different growth indexes and surface response, the optimum concentrations and the (IAA:IBA) ratios can be obtained. The optimum concentrations of IAA and IBA were 167 and 186, 310 and 217, 193 and 159, 191 and 221, and 206 and 186 mg·L^−1^, with corresponding ratios of 1:1.11, 1:0.70, 1:0.82, 1:1.15 and 1:0.90, respectively, at the maximum seedling height and collar diameter growth as well as biomass accumulation at the root, stem and needle. The above growth indexes were 22.00%, 79.80%, 48.65%, 82.20% and 107.00% higher than the control treatment.

## 1. Introduction

Yunnan pine (*Pinus yunnanensis* Franch.), an evergreen arbor of genus *Pinus* and subgenera *Pinus* (of the hard pines, Diploxylon) in the Pinaceae family, is 52 m tall and 1 m in diameter, with a brown-gray colored bark, longitudinally fissured, with 10–30 cm needles in bunches of 3 (rarely 2). It is a common and commercially valuable species in southwestern China, occurring at elevations of 600–3100 m, 23°–30° N and 96°–108° E [1,2]. Seed cones are short, pedunculate, green, maturing to brown or chestnut brown, conical-ovoid, 5–11 cm, dehiscent or indehiscent at maturity. Seed scales are oblong-ellipsoid, 3 × 1.5 cm; apophyses usually swollen, cross keeled, rarely recurved; umbo slightly sunken or slightly protruded, ending in a minute prickle. Seeds are brown, nearly ovoid or obovoid, slightly compressed, 4–5 mm with a wing of 1.6–1.9 cm. Pollination occurs April-May and seed maturity in October of the following year [1,3]. This species plays a crucial role in regional economic development and ecological environment construction [3]. It is also a light-like, deep root, drought-resistant and poor-site tolerant, and may be a pioneer tree species in barren hill afforestation [1]. However, young Yunnan pine seedlings experience slow growth and particularly bad “stagnated growth” within three years after planting [3], which has limited the functioning of economic and ecological benefits of the young growth stage. Obtaining slow growth and accelerating growth in the seedling stage has become the key issue for the Yunnan pine industry. Auxins, a group of phytohormones that have been implicated in most of the quantitative growth changes that occur during a plant’s life cycle [4], play many central roles in the regulation of plant growth and orchestrate many developmental processes of plants and environmental responses [5–8]. Two of the most common auxins in plants are indoleacetic acid (IAA) and indolebutyric acid (IBA), which can promote growth and accelerate cell division [9]. Application of exogenous auxins have various critical effects on plant growth and development, such as directing and controlling cell division, expansion, and differentiation by inducing the positioning of organ primordia and stem cell niches [7,10]; regulating gametophyte induction and growth [11]; manipulating the process of somatic embryo maturation in embryogenic cultures [12]; producing apical dominance, phototropism and gravitropism [13]; promoting root cuttings and increasing the number of roots, forming a strong root system through root growth enhancement and root hair development and elongation [14–17]; and regulating the activities of correlated enzymes and the expression of genes [18–21]. Hadi *et al*. [22] confirmed that IAA can increase plant growth and dry biomass with foliar spray. Little *et al*. [8] showed that IAA appeared to be involved in the promotion of cambial activities and fiber elongation. Singh *et al*. [17] reported that IBA can promote root growth, including root number and length, and collar diameter, which helped the enhancement of the root system. In addition, it has been well established that exogenous IAA promotes vessel development in hardwoods [8]. The above evidence shows that IAA and IBA are recognized as the key auxins in most plants [6]. Many studies have been conducted to determine the effects of a single factor on growth, but relatively few studies have involved two or multiple factors and their interactions. Research has also implicated a role for IAA and IBA in promotion of seedling growth and root cutting formation in Yunnan pine. Li *et al*. [23] investigated the effects of the growth promotion by soaking seeds with IAA and IBA water solution. Seedling height, ground diameter, and biomass accumulation of root, stem, and leaf were 22.64%, 66.12%, 62.40%, 198.00% and 83.30% higher, respectively, than the control. The corresponding dosages of IAA and IBA were 50 and 86, 57 and 144, 53 and 88, 49 and 99, and 45 and 109 mg·L^−1^. Zhao *et al*. [24] reported endogenous IAA (313 mg·L^−1^) and IBA (163 mg·L^−1^) stimulated adventitious root formation in vegetative cutting propagation. However, little is known about the response of the seedling growth of Yunnan pine to exogenous auxin by foliar application.

Therefore, in the present investigation we analyzed the relationship between IAA and IBA treatment with foliar application and the change of growth (increment and biomass) in one-year-old Yunnan pine seedlings. We first confirmed that IAA and IBA can promote seedling growth and additionally we obtained the optimal concentrations and their ratios in their application for the acceleration of Yunnan pine seedling growth.

## 2. Results and Discussion

### 2.1. Trend of Exogenous Auxin Effect

In order to determine whether IAA and IBA could promote Yunnan pine seedling growth, one-year-old seedlings were treated with solutions of different concentrations of IAA in combination with IBA. Based on different experimental combinations, several regression equations were built for each growth index. One optimum regression equation was finally chosen according to correlation coefficient (*R*), significance level and residual standard deviation. Table 1 shows that there exists a significant correlation (*P* < 0.05) between each growth index and the concentration of the IAA and IBA relationship can be described by a quadric surface. There also exists a significant interaction between these two exogenous hormones in the growth of Yunnan pine.

**Figure 1:**
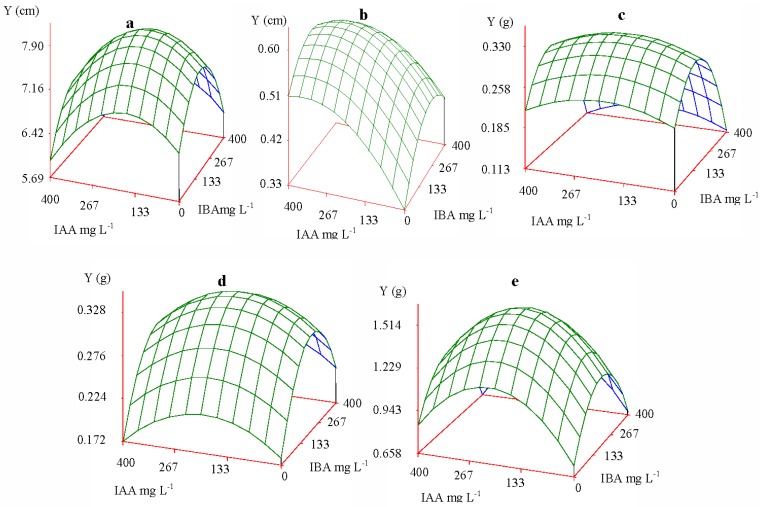
Growth index response of Yunnan pine to exogenous treatment with indoleacetic acid (IAA) and indolebutyric acid (IBA) solution. (**a**) height; (**b**) collar diameter; (**c**) root biomass; (**d**) stem biomass; (**e**) needle biomass.

Figure 1 shows that the effect of IAA and IBA on growth index appeared as a bivariate normal or quadratic parabola surface; that is, each growth index had one maximum peak value. Prior to the peak value, each growth index demonstrated an increase when the concentrations of IAA and IBA were likewise increased; however, after the peak value, each growth index decreased when the concentrations of IAA and IBA were increased. It can be concluded that the growth response and biomass accumulation of Yunnan pine seedlings, in conjunction with the concentration of IAA and IBA, demonstrated conformity with the law of tolerance [25–27]. An appropriate concentration of IAA and IBA could promote seedling growth, while an excessive concentration would inhibit growth. Such dual effects were reported in other investigations. Tong *et al*. [28] found, compared to the control alone, the addition of low concentrations of Fe-chlorophyllin resulted in an increase in the length of wheat roots. However, higher concentrations of Fe-chlorophyllin contributed to inhibition of the growth of wheat roots. Ahmed *et al*. [11] also confirmed that higher concentrations of IAA and IBA inhibited growth. Low and moderate concentrations of growth regulators proved to be most effective [11,29]. In *Arabidopsis thaliana*, Rahman *et al*. [30] reported that application of 10 mM auxin reached the optimum level and 100 mM greatly inhibited the elongation of *Arabidopsis* roots. Ivanchenko *et al*. [31] also verified that lateral root initiation by IAA was reduced as the IAA concentration was increased in the nanomolar range, and IAA became inhibitory at 25 nM. Similar dual effects were reported in other investigations [32–34]. This observation may be explained by the fact that an organism’s tolerance to unfavorable conditions is reduced when other environmental factors are also at non-optimal levels [35,36].

Based on the response of the auxin effect, the quadratic parabola surface vertices were the peak values of increment and biomass in the seedlings, and the corresponding optimum concentrations and ratios can be obtained. The medium concentration of IAA and IBA contributed to height growth and biomass accumulation (root, stem and needle), while high concentrations of IAA and medium concentrations of IBA contributed to the seedlings’ collar diameter growth. Figure 1 shows the effects of auxin on seedling growth at optimum concentrations and ratios. An increase in hormone levels did not lead to increased growth or biomass of the seedlings, and there exists some discrepancy in the optimum concentrations of exogenous auxin and their ratios for different growth indexes.

### 2.2. Pattern of Exogenous Auxin Effects

#### 2.2.1. Pattern of One-Factor Effects

The one-factor effects were analyzed by applying the method of descent, allowing one of the factors to be zero, with each equation possessing only one hormone factor, IAA or IBA. This method produces a quadratic equation, each factor relating to increment and biomass. Table 2 shows each growth index was related to concentration of one exogenous auxin in a parabola curve form, in which the growth index first increased and later decreased when the concentrations of exogenous auxin increased. The growth index increased with the concentration of IAA or IBA before reaching the maximum levels; after reaching maximum levels, it decreased when the concentration of IAA or IBA increased. Table 2 also shows that an application of IAA alone yields maximum increments of height and collar diameter at 6.97 cm and 0.505 cm, which were 7.66% and 52.57% higher compared with the control. The maximum biomass of root, stem and needle were 0.251 g, 0.218 g and 1.161 g, which were 13.10%, 21.10% and 58.60% higher, respectively, compared with the control. When only IBA was applied, the maximum increments of height and collar diameter were 7.40 cm and 0.46 cm, respectively, which were 14.30% and 38.37% higher than the control. The maximum biomass of root, stem and needle were 0.301 g, 0.290 g and 1.120 g, respectively, which were 35.60%, 61.10% and 53.00% higher than the control. The effects of IAA and IBA on the increment and biomass of Yunnan pine seedlings can be described by a parabola curve. Each growth index had an optimum concentration of hormone. The application of appropriate concentrations of IAA and IBA can accelerate seedling growth and biomass accumulation, while excessive concentrations of IAA and IBA had an inhibitory effect. Such similar regularities were also confirmed in other investigations. Recently, Gangwar *et al*. [37,38] found an addition of 10 μM IAA alleviated negative effects of metal toxicity symptoms on growth of legume species; however, the addition of 100 μM yielded the opposite response. Such regularity of tolerance was conformed in the present study.

#### 2.2.2. Pattern of Interaction of IAA and IBA

An interaction effect analysis was also performed using the method of descent; it differed from the one-factor effect analysis and also involved two factors of both IAA and IBA simultaneously. From the analysis we found that the optimum concentration of IAA and IBA with foliar spray for Yunnan pine seedlings and their ratios, the theoretical maximum yield, and the related parameters can be obtained. Table 3 shows the interaction and optimum ratio of IAA and IBA. The theoretical maximum increments of height and collar diameter were 7.90 cm and 0.60 cm, respectively, which were 13.34% and 17.80% higher than that of applying only IAA, 6.76% and 29.90% higher than that of applying only IBA, and 22.00% and 79.80% higher than the control. The theoretical maximum biomasses of root, stem and needle were 0.330 g, 0.328 g and 1.516 g, which were 31.50%, 50.50% and 30.60% higher than that of applying only IAA, 9.60%, 13.10% and 35.40% higher than that of applying only IBA, and 48.65%, 82.20% and 107.00% higher than the control. A higher proportion of IAA accelerated diameter growth of the stem, development of the root system and the accumulation of leaf biomass, while higher proportions of IBA contributed to the stem’s height growth and biomass accumulation. The optimum concentration of IAA and IBA was just within the range of experimental design, which ensured the precision of the experiment. It can be seen that the interaction effect of the application of IAA in combination with IBA was higher than that of applying only IAA or IBA alone. However, the response of different growth indexes to the concentration of the two hormones and their ratios was different to some degree.

#### 2.2.3. Simulation Test of Whole Factors

Two factors of IAA and IBA and three levels of each factor had 9 different tests. When they were put into the hormone effect equation, this can simulate 9 results (Table 4). Table 4 shows that when only IAA (Treatment 1, 4 and 7) was applied and IBA alone was applied (Treatment 1, 2 and 3), each growth index increased first and then decreased with an increase in the concentration of IAA and IBA, which can be described by a parabola curve. The increment and biomass had the highest values when applying the medium concentration of IAA or IBA (Treatment 4 and 2). The highest increment and biomass were a result of the combination of two hormones (Treatment 5, 6, 8 and 9), but also a result of applying the medium concentration (Treatment 5). The increments of both height and collar diameter, and biomass of root, stem and needle were 21.79%, 74.00%, 46.40%, 81.67% and 106.80% higher compared to the control (Treatment 1), 13.38%, 21.52%, 29.48%, 50.00% and 30.52% higher than when IAA was applied alone, and 6.63%, 26.59%, 9.80%, 13.15% and 35.18% higher than when only IBA was applied. These effects were followed by the medium concentration of IAA and the highest concentration of IBA (Treatment 6), and the highest concentration of IAA and a medium high concentration of IBA (Treatment 8). After the application of the highest concentrations of both IAA and IBA (Treatment 9), 3 growth indexes (with the exception of the collar diameter increment and stem biomass) were lower than those of the control (Treatment 1). It can be seen that the application of an appropriate concentration of IAA and IBA could significantly improve the growth of Yunnan pine seedlings; however, an excessive concentration of hormones could inhibit seedling growth.

By summarizing the application results of only the hormones applied alone and then in combination, we see that both the increment and biomass of Yunnan pine seedlings increased first and then decreased with an increase of hormone concentration, regardless of whether that hormone was IAA (Treatment 1, 4 and 7) or IBA (Treatment 1, 2 and 3), or a combination of the two (Treatment 5, 6, 8 and 9). The application of combined IAA and IBA had better effects than the hormones applied independently, and the combination had an optimum concentration and ratio.

### 2.3. Optimum Concentrations and Ratios of IAA and IBA

**Figure 2:**
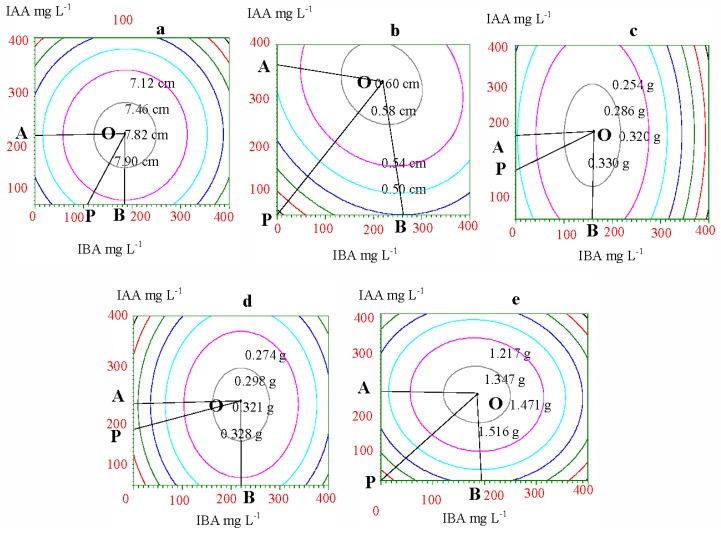
Yield-equality lines of growth and biomass in response to IAA and IBA. (**a**) height; (**b**) collar diameter; (**c**) root biomass; (**d**) stem biomass; (**e**) needle biomass.

To gain the optimum concentrations and ratio of IAA and IBA, the same-height points (yields) were connected on each response surface and an equal yield line was gained. Vertical projections of these equal yield lines show graphs that correspond to the response surface. As Figure 2 shows, each equal yield line was distributed in concentric circles, and the circle’s central point showed the maximum yield corresponding to the optimum concentration of hormones and their ratios. Any two points on each equal yield line had the same yields, although they represented a different concentration of IAA and IBA. Therefore, we can find the single point on the same equal yield line that had the minimum concentration of IAA and IBA or the least investment for the same yield. This point represents the most economic concentration of IAA and IBA. The area covered by the Crest line OA and OB and coordinate axis was the rational concentration hormone range (Figure 2, Table 5), and the concentration of IAA and IBA beyond this area would not increase the yield but rather the investment. The OP line was the optimum concentration and ratio of IAA and IBA under the hormone price limit. Taking increments of collar diameter as an example (Figure 2b and Table 5), the rational concentration of IAA and IBA were 0~352.11 mg·L^−1^ and 0~261.78 mg·L^−1^, respectively. Therefore, the optimum concentrations of IAA and IBA, and their optimum ratios, can be gained from the OP line equation.

## 3. Experimental Sections

### 3.1. Plant Material

The seeds used in this experiment were harvested from a superior open-pollinate mature tree, *Pinus yunnanensis*, from Clonal Seed Orchard in Midu, Dali State, Yunnan Provence, China (latitude: 25°27′N; longitude: 100°28′; elevation: 1900 m). The 1000 kernel weight of seed, field germination energy and germination rate was 16.99 g, 50% and 90%, respectively. The seeds were sterilized with 0.1% HgCl_2_, rinsed several times with distilled water, soaked in distilled water for 12 h and placed in a plastic film seedling container (13 cm × 17 cm) filled with soil substrate at the Lufeng Village Forest Farm of Yiliang County in Kunming, Yunnan, China (latitude: 24°30′N; longitude: 103°08′; elevation: 1800 m). There was one seed in each container. When the seedlings were two months old, they were selected for uniformity with a height and diameter of 2.85 ± 0.10 cm and 0.12 ± 0.01 cm, respectively. They were pooled and randomly distributed in each plot.

### 3.2. Phytohormone Preparation

The exogenous auxins IAA (indole-3-acetic acid) and IBA (indole-3-butyric acid) were purchased from Huishi Biochemical Ltd., Shanghai, China. They are analytical reagent (AR) grade. IAA was a white powder, with more than 98% purity; IBA was light grey, with more than 99% purity. The molecular weights were 175.19 and 203.24 Da, respectively. They were dissolved in a little alcohol, diluted with distilled water and brought to a solution of 1 g L^−1^. The different treatment solutions (Table 6) were obtained through mixing IAA, IBA solution, and distilled water.

### 3.3. Experimental Design

The experiment used a 3 × 3 regression design with 2 factors and 3 levels. The concentration of IAA and IBA were selected according to the Sen B.R. [39] method, and three concentration levels of high, medium and low were chosen. Level High was two times the Level Medium concentration, and the concentration in Level Low was 0 (as control). The concentration of both IAA and IBA used in Level High was 400 mg·L^−1^. The experiment scheme is shown in Table 6.

The experiment’s field arrangement has been optimized based on the characteristics of a Latin square. The experiment had three replications. Each replication consisted of one block composed of 9 plots (for details, see Table 7). The complete experiment contained 27 plots, with 30 seedlings per plot (*n* = 810 seedlings: 30 seedlings × 9 plots × 3 replicates). When seedlings were two-months-old, an IAA and IBA water solution was sprayed on the needles with a microsprayer until the needles were hung over solution but not dropped. Foliar applications were performed once every 5 days and repeated five times.

### 3.4. Data Collection and Statistical Analysis

Increments were measured for every seedling, and was performed after seedlings ended their current-year growth at a time when the seedlings were one-year-old.

Biomass was measured by using a mean sample tree [40,41] method, in which the biomass of root, stem and needle of one mean sample tree was collected from each plot after the seedling ended its current-year growth, and was measured in the lab, by using the thermostatic drying oven method.

Based on the regression analysis of experimental data, a hormone effect model could be built:

(1) $$Y=a_{0}+a_{1}A+a_{2}B+a_{3}A^{2}+a_{4}B^{2}+a_{5}AB$$

where, *Y* is growth index; *a*_0_, *a*_1_, *a*_2_, *a*_3_, *a*_4_, and *a*_5_ are coefficients to be determined; *A* is concentration of IAA, *B* is concentration of IBA, *AB* is interaction between IAA and IBA.

From this equation, we can analyze one factor and interaction effect by using the method of descent [42], and find the optimum hormone concentration and their ratio, range of appropriate hormone concentration, and minimum cost line.

## 4. Conclusions

Spraying the Yunnan pine seedlings with IAA and IBA can significantly improve their growth and biomass accumulation, which confirms this as an effective way to solve their slow growth problems during the early stage of development. The application of IAA combined with IBA had better results than the application of only one hormone, but the results did not show higher seedling growth in proportion with increased hormone concentrations. The response of seedlings’ growth and biomass accumulation in relation to the concentration of hormones applied can be described by a quadratic surface response; both their growth and biomass increased first, then decreased with increasing hormone levels, and each growth index had a peak value. The growth index increased with the increase of IAA and IBA concentrations before reaching peak growth values, then decreased. Based on the auxin effect equations of the different growth indexes, the optimum concentrations and ratios of IAA and IBA can be obtained at the maximum yield. The optimum concentrations of exogenously applied IAA and IBA for maximum height, collar, root, stem and needle growth were 167 and 186, 310 and 217, 193 and 159, 191 and 221, and 206 and 186 mg/L, respectively. The optimum ratios of IAA to IBA for maximum height, collar, root, stem and needle growth were 1:1.11, 1:0.70, 1: 0.82, 1:1.15 and 1:0.90, respectively. According to the high genetic correlations among growth-curve parameters [43], the early growth promotion was effective.

## Figures and Tables

**Table 1:** Auxin effect equations for the growth index of Yunnan pine.

Growth index(*Y*)		Auxin effect equations	Correlation coefficient (*R*)	*F* value	Significance level (*P*)
Increment	Height	$Y=6.474+0.006A+0.01B-0.0000182A^{2}-0.0000271B^{2}+0.000000375AB$	0.987	22.619	0.014
	Collar diameter	$Y=0.331+0.001A+0.001B-0.000000142A^{2}-0.00000191B^{2}-0.00000055AB$	0.975	11.541	0.036
Biomass	Root	$Y=0.222+0.0003A+0.001B-0.00000783A^{2}-0.00000316B^{2}+0.0000000125AB$	0.991	32.891	0.008
	Stem	$Y=0.18+0.0004A+0.001B-0.00000105A^{2}-0.00000227B^{2}+0.00000000625AB$	0.970	9.532	0.046
	Leaf	$Y=0.732+0.004A+0.004B-0.0000093A^{2}-0.0000103B^{2}-0.00000086AB$	0.822	7.476	0.001

Note: A and B represent the concentration levels of IAA and IBA, respectively, and AB is the interaction between the two exogenous hormones.

**Table 2:** One-factor equations of IAA and IBA on the growth index of Yunnan pine seedlings.

Growth index(*Y*)		One-factor equation	Maximum yield	One-factor equation	Maximum yield
Increment	Height	$Y=6.474+0.006A-0.0000182A^{2}$	6.97 cm	$Y=6.474+0.01B-0.0000271B^{2}$	7.40 cm
	Collar diameter	$Y=0.331+0.001A-0.00000142A^{2}$	0.51 cm	$Y=0.331+0.001B-0.00000191B^{2}$	0.46 cm
Biomass	Rroot	$Y=0.222+0.0003A-0.000000783A^{2}$	0.251 g	$$Y=0.222+0.001B-0.00000316B^{2}$$	0.301 g
	Stem	$Y=0.18+0.0004A-0.00000105A^{2}$	0.218 g	$$Y=0.18+0.001B-0.00000227B^{2}$$	0.290 g
	Leaf	$Y=0.732+0.0004A-0.0000093A^{2}$	1.161 g	$Y=0.732+0.004B-0.0000103B^{2}$	1.120 g

Note: *A* and *B* are the concentration of IAA and IBA.

**Table 3:** Interaction effect analysis of growth index in response to IAA and IBA.

Growth index (*Y*)		Optimum concentration		Optimum ratio of IAA and IBA IAA:IBA	Theoretical maximum yield

		IAA mg·L^−1^	IBA mg·L^−1^		
Increment	Height	166.75	185.66	1:1.11	7.90 cm
	Collar diameter	310.06	217.14	1:0.70	0.60 cm
Biomass	Root	192.84	158.61	1:0.82	0.330 g
	Stem	191.13	220.53	1:1.15	0.328 g
	Leaf	206.47	185.55	1:0.90	1.516 g

**Table 4:** Simulation test results of growth index in response to IAA and IBA.

Treatment number.	IAA mg·L^−1^	IBA mg·L^−1^	Increment		Biomass		

			Height (cm)	Diameter (cm)	Root (g)	Stem (g)	Leaf (g)
1	IAA_0_ (0)	IBA_0_ (0)	6.47	0.33	0.222	0.180	0.732
2	IAA_0_ (0)	IBA_1_ (200)	7.39	0.46	0.296	0.289	1.120
3	IAA_0_ (0)	IBA_2_ (400)	6.14	0.43	0.116	0.217	0.684
4	IAA_1_ (200)	IBA_0_ (0)	6.95	0.47	0.251	0.218	1.160
5	IAA_1_ (200)	IBA_1_ (200)	7.88	0.58	0.325	0.327	1.514
6	IAA_1_ (200)	IBA_2_ (400)	6.64	0.53	0.146	0.266	1.043
7	IAA_2_ (400)	IBA_0_ (0)	5.96	0.50	0.217	0.172	0.844
8	IAA_2_ (400)	IBA_1_ (200)	6.91	0.58	0.291	0.282	1.163
9	IAA_2_ (400)	IBA_2_ (400)	5.69	0.51	0.113	0.210	0.658

**Table 5:** Tangency points of yield-equality lines graph and op-curve equations of growth in response to IAA and IBA.

Growth index(*Y*)		Tangency point of ridgeline mg·L^−1^ (Point *A*, *B*)		OP line equation	Highest yield (Point *O*)

		OA	OB		
Increment	Height	IAA = A = 164.84	IBA = B = 184.50	0.0000546A-0.0000368B+0.004=0	7.90 cm
	Collar Diameter	IAA = A = 352.11	IBA = B = 261.78	0.00000229B-0.00000327A=0	0.60 cm
Biomass	Root	IAA = A = 191.57	IBA = B = 158.23	0.00000633A+0.00000158B-0.0007=0	0.330 g
	Stem	IAA = A = 190.48	IBA = B = 220.26	0.00000455A+0.00000211B-0.0006=0	0.328 g
	Leaf	IAA = A = 215.05	IBA = B = 194.17	0.0000177B-0.0000197A=0	1.516 g

**Table 6:** The experimental design of IAA and IBA.

Treat number	1	2	3	4	5	6	7	8	9
Concentration of IAA mg·L^−1^	0	0	0	200	200	200	400	400	400
Concentration of IBA mg·L^−1^	0	200	400	0	200	400	0	200	400

**Table 7:** Field arrangement of IAA and IBA.

Item	Field test arrangement								
Replication I	1	2	3	4	5	6	7	8	9
Replication II	4	5	6	7	8	9	1	2	3
Replication III	7	8	9	1	2	3	4	5	6
